# Systematic analysis of enhancer regulatory circuit perturbation driven by copy number variations in malignant glioma

**DOI:** 10.7150/thno.54150

**Published:** 2021-01-01

**Authors:** Jun Xiao, Xiaoyan Jin, Chunlong Zhang, Haozhe Zou, Zhenghong Chang, Nan Han, Xia Li, Yunpeng Zhang, Yongsheng Li

**Affiliations:** 1 Key Laboratory of Tropical Translational Medicine of Ministry of Education, College of Biomedical Information and Engineering, Hainan Medical University, Haikou 571199, China; 2 College of Bioinformatics Science and Technology, Harbin Medical University, Harbin 150081, China

**Keywords:** Enhancer, regulatory circuit, copy number variation, prognostic, glioma progression

## Abstract

**Background:** Enhancers are emerging regulatory regions controlling gene expression in diverse cancer types. However, the functions of enhancer regulatory circuit perturbations driven by copy number variations (CNVs) in malignant glioma are unclear. Therefore, we aimed to investigate the comprehensive enhancer regulatory perturbation and identify potential biomarkers in glioma.

**Results:** We performed a meta-analysis of the enhancer centered regulatory circuit perturbations in 683 gliomas by integrating CNVs, gene expression, and transcription factors (TFs) binding. We found widespread CNVs of enhancers during glioma progression, and CNVs were associated with the perturbations of enhancer activities. In particular, the degree of perturbations for amplified enhancers was much greater accompanied by the glioma malignant progression. In addition, CNVs and enhancers cooperatively regulated the expressions of cancer-related genes. Genome-wide TF binding profiles revealed that enhancers were pervasively regulated by TFs. A network-based analysis of TF-enhancer-gene regulatory circuits revealed a core TF-gene module (58 interactions including seven genes and 14 TFs) that was associated survival of patients with glioma (p < 0.001). Finally, we validated this prognosis-associated TF-gene regulatory module in an independent cohort. In summary, our analyses provided new molecular insights for enhancer-centered transcriptional perturbation in glioma therapy.

**Conclusion:** Integrative analysis revealed enhancer regulatory perturbations in glioma and also identified a network module that was associated with patient survival, thereby providing novel insights into enhancer-centered cancer therapy.

## Introduction

Glioma is one of the most common brain tumors 1, 2, which is classified into grades I-IV according to the World Health Organization (WHO) 3. Accumulating evidence suggests that perturbation of gene expression is closely associated with carcinogenesis, which might be triggered by genomic rearrangement 4, site-specific transcription factors 5, or long-range enhancer interactions 6. However, the molecular basis of glioma pathogenesis is still poorly understood.

With the development of high-throughput sequencing technologies, it has been proven that DNA copy number aberrations (CNAs) play an important role in cancer biology 7, 8. Gene CNAs may impact the activities of a variety of oncogenic or tumor-suppressive pathways 9-11. However, CNAs located outside the gene regions also affect the expression of genes *via* an indirect mechanism. Our knowledge about the effects of these CNAs on gene expression at a genome-wide level is still limited. Enhancers are a well-known class of regulatory regions 12-14, and they have been shown to control genes important for maintaining cellular identity. CancerEnD is a comprehensive resource recently developed for annotating expressed enhancers and associated genes across cancer types 15. Perturbations of enhancer activities are also frequently associated with oncogenes and translocations that result in aberrant gene expression in cancer 16. Paul *et al.* reported that gains of CNAs impact the enhancer activation in medulloblastoma, which might be responsible for overexpression of target gene GFI1B 17. Gene copy number amplification can also lead to chemotherapy resistance and poor overall survival in patients with cancer 11, 18. Copy number gains of non-coding regions harboring super-enhancers were found near four known cancer-related genes in human epithelial cancers 19. However, a global view of enhancer activity alterations during glioma progression remains poorly understood.

Moreover, the transcriptional activity of genes is regulated through the interplay between enhancers and other *cis*-acting regulatory elements bound by transcription factors (TFs) 20. Recent studies have shown that TFs play critical roles in the regulation of enhancer activation and, thus, control enhancer function 21. For example, tumor suppressor p53 regulates enhancer accessibility and activity in response to DNA damage 22. Under the regulation by TFs, enhancers usually perform their functions through targeting upstream and downstream genes. Therefore, genome-wide identification and characterization of the regulatory relationships between enhancers, TFs and target genes during glioma progression are necessary to reveal underlying regulatory circuit perturbations in enhancer regulatory contexts.

In this study, we performed a meta-analysis of the relationship between TFs, enhancers, and target gene networks, and a comprehensive functional dissection of enhancer circuits during glioma progression. By integrative analysis of the CNAs and enhancer data, widespread CNAs of enhancers were observed during glioma progression. In addition, CNAs and enhancers cooperatively regulated the expression of target genes. Importantly, a network-based analysis of TF-enhancer-gene regulatory circuits revealed a core TF-gene module that was associated with glioma prognosis.

## Methods

### CNA and gene expression data during glioma progression

We obtained the level-3 copy number variation (CNV) data of Affymetrix SNP 6.0 array for both low-grade glioma (LGG) and glioblastoma multiforme (GBM) from The Cancer Genome Atlas (TCGA) data portal 23. The patients with LGG (n = 529) were divided into grade II and grade III glioma based on clinical information downloaded from the TCGA project. As a result, there were 258 patients with grade II, 271 patients with grade III and 154 patients with grade IV glioma with CNV data, respectively.

RNA-Seq based gene expression data for patients with glioma (LGG and GBM) were also downloaded from the TCGA data portal. The expression levels of genes were measured as fragments per kilobase of transcript per million fragments mapped reads (FPKM). There were also five normal samples. We obtained 224, 233, and 146 samples with both the CNV and expression data in grade II, III, and IV gliomas, respectively.

### Identification of enhancers with CNV alterations in glioma

**Figure 1:**
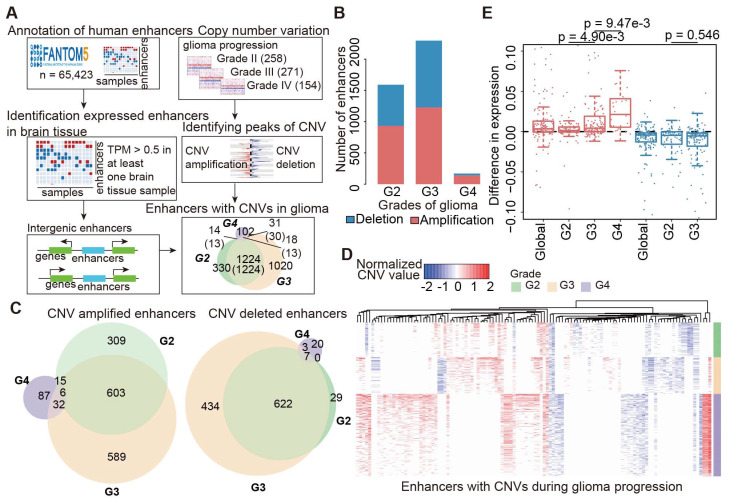
** Widespread CNAs of enhancers during glioma progression.** (A) Flowchart showing identification of enhancers with CNAs in different grades of glioma patients. (B) Numbers of enhancers with CNAs in G2, G3 and G4 glioma patients. Red for enhancers with copy number amplification, and blue for enhancers with copy number deletion. (C) Venn plots showing the overlap of enhancers with CNVs in G2, G3 and G4. (D) Heat map showing the normalized CNVs of enhancers during glioma progression. (E) Boxplots showing the differences of enhancer expression between amplified/deleted and other glioma patients.

To identify the enhancers with CNV alterations in glioma, we first downloaded 65,423 human enhancers from FANTOM5 data portal 24. The enhancer activity in FANTOM5 was detected by CAGE-Seq (Cap Analysis of Gene Expression). The expressions of enhancers were measured by transcripts per kilobase million (TPM). The expression level of an enhancer has been used as an index of its activity 25. Therefore, we removed enhancers with no expression in brain tissue samples (Figure 1A). Moreover, we limited enhancers to those overlapped within intronic or intergenic regions but not promoters (TSS ± 2 kb) or exon regions, resulting in a subset of intronic and intergenic enhancers. In total, 27,165 enhancers were identified in brain tissue.

Next, GISTIC was used to identify recurrent CNV regions in glioma and peaks with confidence window 0.95 were identified 26. Both CNV amplified and deleted regions were identified during glioma progression. We hypothesized that the activity of an enhancer would be increased or decreased if it overlapped with a CNV amplified or deleted region. Thus, BEDtools was used to assign enhancers to CNVs-associated peak regions 27. In addition, the CNV status of enhancers in each glioma sample was obtained for characterizing the activity alterations.

To validate the enhancers with CNVs alteration in glioma tissues, we also obtained the CNVs of cell lines from Cancer Cell Line Encyclopedia (CCLE). Cell lines annotated as “Central_nervous_system” were used. GISTIC was used to identify recurrent CNV regions in cell lines. Peaks with confidence window 0.9 were identified. Next, BEDtools was used to assign enhancers to CNV-altered regions. We used the hypergeometric test to evaluate the overlap of enhancers between the cell lines and glioma tissues.

### Enhancer-target gene regulation in glioma

Identification of target genes of enhancers is critical for understanding their function in cancer. We next predicted the enhancers' potential target genes by considering the distance and expression between enhancers and protein-coding genes. First, we identified the nearest protein coding gene located within 1 kb to 10 Mb from an enhancer. This process was performed using BEDtools 27.

Next, we evaluated the expression correlation between enhancers and these genes. Patients with glioma were divided into two groups depending on the presence of CNVs of an enhancer (the group with CNAs of an enhancer, and the group without CNAs). Gene expression differences between the two groups were evaluated by *t*-test. Genes with a false discovery rate (FDR) < 0.05 were identified as potential target genes of enhancers. Moreover, we looked for genes showing higher expression in enhancer-amplified patients or lower expression in enhancer-deleted patients. After iterations for all enhancers, we identified enhancer-target gene regulation in glioma.

### Identification of TF-enhancer-target gene regulatory circuit in glioma

For the prediction of TF-binding sites on enhancers, we uploaded the coordinates of enhancers to UCSC Table Browser to obtain the sequences. All the sequences of enhancers with FASTA format were downloaded. We next screened the sequences of enhancers for potential TF-binding sites by Match (TM) in TRANSFAC Professional (release 2013.6). Only human TF-binding sites were screened. We identified all enhancers with at least one TF-binding site. To identify TFs that significantly targeted these enhancer sequences, we first obtained TF motifs from TRANSFAC professional. The AME tool integrated in MEME Suite was used to identify TF motifs that were significantly enriched in CNV-altered enhancers compared with all enhancers provided by FANTOM5 28, 29. TF motifs with p < 0.005 were identified as enriched motifs.

We integrated TF-enhancer regulation and enhancer-target interactions identified above to identify all TF-enhancer-target gene triplets. Moreover, we calculated the Spearman correlation coefficient (SCC) between the expression of TFs and target genes. The triplets with co-expressed TF-gene (absolute SCC > 0.3 and FDR < 0.01) were kept for further analysis.

Next, we used *t* test to identify the TFs and target genes showing differential expression between cancer and normal tissues. TFs and genes with FDR < 0.1 and > 1.2-fold changes were identified as differentially expressed. The expression of TFs and target genes were positively correlated if both of them were upregulated or downregulated in glioma. Otherwise, the expression of TFs and target genes had a negative correlation if they showed the opposite directions in glioma. All of the TF-genes were assembled into a gene regulatory network in glioma. The network was visualized by Cytoscape 3.6.1 30, 31.

### Functional enrichment analysis

A cumulative hypergeometric test was used to identify the significantly overrepresented biological function categories or pathways for a gene set of interest. All human genes were considered as background gene set in this analysis. Gene Ontology (GO) term and Kyoto Encyclopedia of Gene and Genome (KEGG) pathways were considered.

### Survival analysis

All patients with glioma were divided into a training set and a testing set of similar gender and age. We next used the Cox hazard analysis to identify the genes associated with survival 32, 33. Genes with p < 0.05 were identified based on the expression profiles in the training set. We calculated the risk score for a patient with glioma* i* as follows:


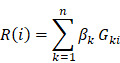


where *n* is the number of genes identified in the Cox regression analysis. 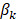 is the regression coefficient for gene *k*, and 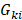 is the expression of gene *k* in patient *i*. Patients in the training set were divided into low-risk and high-risk groups based on the median of the risk scores. In the testing set, we used the same regression coefficients generated in training set to calculate the risk scores. The same threshold as in the training set was used to classify patients into low-risk and high-risk groups. Log-rank test was used to compare the survival between low-risk and high-risk patients.

### Cell lines and reagents

U251 cells were obtained from the American Type Culture Collection (ATCC, Manassas, Virginia, USA). All cells were cultured at 37°C in a humidified incubator with 5% CO_2_ in Dulbecco's modified Eagle's medium (DMEM) (Invitrogen, Carlsbad, CA, USA) with 10% fetal bovine serum (FBS) (HyClone, Logan, UT, USA), 100 U/mL penicillin and 100 μg/mL streptomycin (Invitrogen), and 8 mg/l antibiotic tylosin tartrate against mycoplasma (Sigma-Aldrich, St. Louis, Missouri, USA). Cell lines were authenticated by short tandem repeat (STR) profiling and confirmed to be mycoplasma-free. The cell line was used within 20 passages and subjected to routine cell line quality examinations (e.g., morphology, mycoplasma), and thawed fresh every two months.

### RNA interference

The sequences of small interfering RNA (siRNA) oligonucleotides targeting MAML2/FAM84B/CDK6 and the negative control siRNA were purchased from RiboBio (RiboBio Biotechnology, Guangzhou, China). Transfections with siRNA (75 nM) were performed with Lipofectamine 2000. The sequences of siRNAs were provided in Table S1.

### Reverse transcription PCR and quantitative real-time PCR

RNA samples from the U251 cell line were extracted with TRIzol reagent (Invitrogen). First-strand cDNA was synthesized using the PrimeScript™ Reverse Transcriptase kit (Takara, Dalian, China). Relative RNA levels determined by quantitative real-time PCR (qPCR) were measured on a 7900 Real-Time PCR System with the SDS 2.3 software sequence detection system (Applied Biosystems, USA) using the SYBR Green (Takara) method. β-actin was employed as an internal control. The relative levels of RNA were calculated using the comparative CT (2^-ΔΔ^CT) method.

### Cell proliferation assay

siNC and siRNA U251 cells were seeded in 96-well flat-bottomed plates, with each well containing 1500 cells in 100 μL of cell suspension. After a certain time in culture, cell viability was measured using Cell Counting Kit-8 (CCK-8) assay (Dojindo, Kumamoto Prefecture, Japan). Each experiment with six replicates was repeated three times.

### Migration assay

The migration assay was conducted similar to our previous studies 18, 34, without coating the filters with Matrigel. The cells (5 × 10^4^) were added to the coated filters in a serum-free medium. We added DMEM containing 10% FBS to the lower chambers as a chemoattractant. After 24 h at 37°C in an incubator at 5% CO_2_, the cells that migrated through the filters were fixed with methanol and stained with crystal violet. The cell numbers were counted in five random fields.

## Results

### Widespread CNAs of enhancers during glioma progression

CNAs and somatic mutations are common types of genomic alterations in cancer. To explore the relationship between genomic instability and enhancers during glioma progression, we proposed a computational pipeline to determine CNV-driven enhancers (Figure 1A). Particularly, we focused on intergenic enhancers expressed in brain tissues. In total, we identified 27,165 active enhancers in brain tissue out of 65,423 FANTOM enhancers.

Next, we applied GISTIC 2.0 to Affymetrix SNP6.0 arrays in TCGA LGG and GBM cohorts. We found 226, 193, and 367 amplification peaks and 309, 249, and 317 deletion peaks in patients with grade II, grade III and grade IV glioma, respectively. Similar to a previous study 35, we found that CNAs were frequently observed in chromosomes 1, 7, and 10 (Figure S1). Moreover, the CNAs were more complex and prevalent during glioma progression, and patients with high-grade glioma were likely to harbor more CNAs (Figure S1A-C).

To identify enhancers implicated by CNAs, we examined the overlap of the CNAs-associated peak regions and active enhancers in brain tissue. A total of 933, 1,230, and 140 enhancers located in 26, 32, and 46 amplification peaks and 658, 1,066, and 30 enhancers located in 25, 22, and 22 deletion peaks were identified in patients with grade II, grade III and grade IV glioma, respectively (Figure 1B). Moreover, we found that there were more CNA-driven enhancers shared between grades II and III, and more specific enhancers (87 amplified and 20 deleted) were specifically altered in grade IV (Figure 1C and Figure S1D). We also identified 18 enhancers shared in all grades, including six consistently amplified and seven consistently deleted enhancers (Figure 1D and Figure S1D). Next, we identified the CNA-driven enhancers in cell lines of the central neural system. We found that a significantly overlap of the CNA-driven enhancers between the cell lines and glioma tissues (Figure S2, p < 0.001 for amplification and deletion), demonstrating the predicted amplification or deletion of enhancer regions in glioma. Taken together, those results presented a general picture of enhancer alterations (1,641 amplified and 1,115 deleted enhancers in total) during glioma progression and suggested widespread CNAs of enhancers, complementary to other genomic elements.

### CNAs are associated with enhancer activation

To explore the relationship between genomic instability and enhancer expression during glioma progression, we overlapped the active enhancers in the brain to 15,808 enhancers identified in a recent study 36. The expression data of enhancers retrieved from RNA-Seq in the TCGA LGG and GBM cohorts were downloaded. Moreover, we considered only enhancers that were expressed in at least 10% of samples. Previous studies have demonstrated that an obvious consequence of copy number changes is alteration in gene dosage 37, 38, which results in perturbations of gene expression. Therefore, we explored whether the expression levels of enhancers were perturbed by CNAs during glioma progression. We found that enhancer RNAs (eRNAs) were upregulated for enhancers located in CNV-amplified regions (Figure 1E and Figure S1E). In contrast, enhancers that located in CNV-deleted regions exhibited downregulated expression trend in gliomas. Moreover, the expression difference for amplified enhancers was much greater during the glioma malignant progression (Figure 1E). Collectively, all these results suggested the association of CNAs with enhancer activation during glioma progression.

### CNAs of enhancers perturb expression of cancer-related target genes

**Figure 2:**
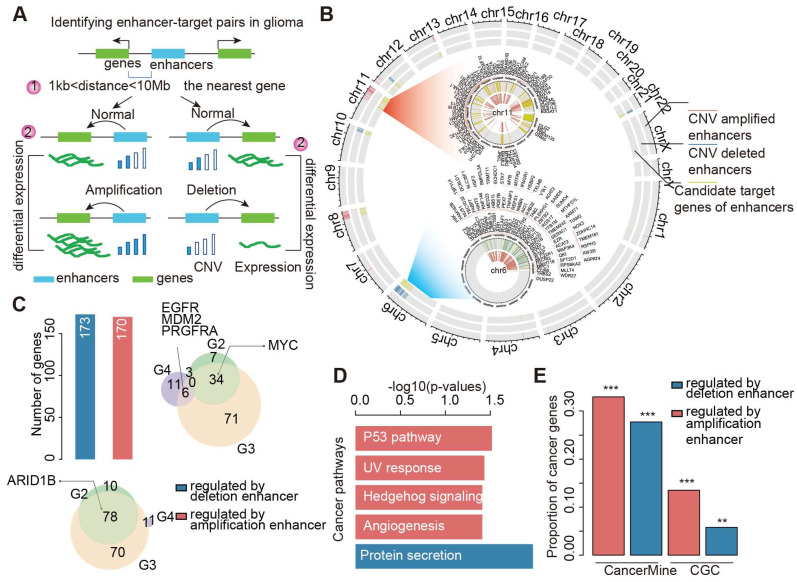
** CNAs of enhancers regulate cancer-related genes.** (A) The framework for identifying genes potentially regulated by enhancers. (B) Circos plots showing the enhancer-gene regulation in glioma. Red regions for enhancers with copy number amplification, blue regions for enhancers with copy number deletion, and green regions for genes regulated by CNV-driven enhancers. (C) Numbers of genes regulated by enhancers in glioma. Bar plots showing the number of genes. Venn plots showing the overlaps of target genes regulated by amplified or deleted enhancers. (D) Pathways enriched by target genes of enhancers. Red for genes regulated by amplified enhancers and blue for genes regulated by deleted enhancers. (E) Proportion of cancer genes in target genes regulated by enhancers. The significance levels were calculated by comparison with randomly selected genes.

To investigate the potential functions of enhancers in cancer development and progression, it is critical to identify their downstream target genes. By integration of genomic locations and expression profiles, we predicted the potential candidate target genes of enhancers in two steps (Figure 2A). In total, we identified 170 genes showing higher expression in enhancer-amplified patients, and these genes were involved in 678 interactions with enhancers (Figure 2B and Figure S3). By contrast, 173 genes involved in 565 interactions showed lower expression in enhancer-deleted patients. In particular, we found that the interactions of the amplified enhancers were mainly located on chromosomes 8 and 11 (Tables S2 and S3). The interactions of deleted enhancers primarily occurred on chromosomes 6 and 10 (Figure 2B and Figure S3).

We next repeated this process for each grade of glioma and found that these enhancers regulated a number of cancer-related genes, such as ARID1B 39, EGFR, MDM2 40, PRGFRA, and MYC (Figure 2C). For instance, EGFR has been demonstrated as a clinical marker in glioblastoma 41. We found that it was regulated by two enhancers (E1 and E2, Figure S4A). E1 was amplified in grade III, and E2 was amplified in both grade III and grade IV patients. We investigated the expression of E1 and found significantly higher expression in amplified patients (Figure S4B, p = 2.12e-4). Moreover, we found significantly higher EGFR expression in patients with E1- and E2-amplified glioma (Figure S4C-D). Another example is the MYC gene, we found seven enhancers with increased activity regulating MYC in both grades II and III (Figure S5). The majority of these enhancers were located in super-enhancer deriving from brain tissue-SF268 42.

To investigate the function of genes regulated by enhancers, we performed functional enrichment analysis. We found that these genes were significantly enriched in P53 pathway, ultraviolet (UV) response, angiogenesis and protein secretion (Figure 2D). These results suggested that genes regulated by enhancers were likely to be involved in cancer. We next explored to what extent these genes were validated as cancer-related genes. Thus, we calculated the proportion of cancer genes among the amplified or deleted enhancer-regulated genes. Approximately 30% of genes were curated in CancerMine 43, while 15% of genes regulated by amplified enhancers were annotated as known cancer genes in Cancer Gene Census 44 (Figure 2E). We next randomly selected the same number of genes as enhancer regulated genes and recalculated the proportion of cancer-related genes. This process was repeated 10,000 times. We found that genes regulated by enhancers were more likely to be associated with cancer (Figure 2E, p < 0.05). Together, all these results suggested that CNAs of enhancers perturbed the expression of cancer-related genes.

### Enhancers and CNAs cooperatively regulate expression of target genes

**Figure 3:**
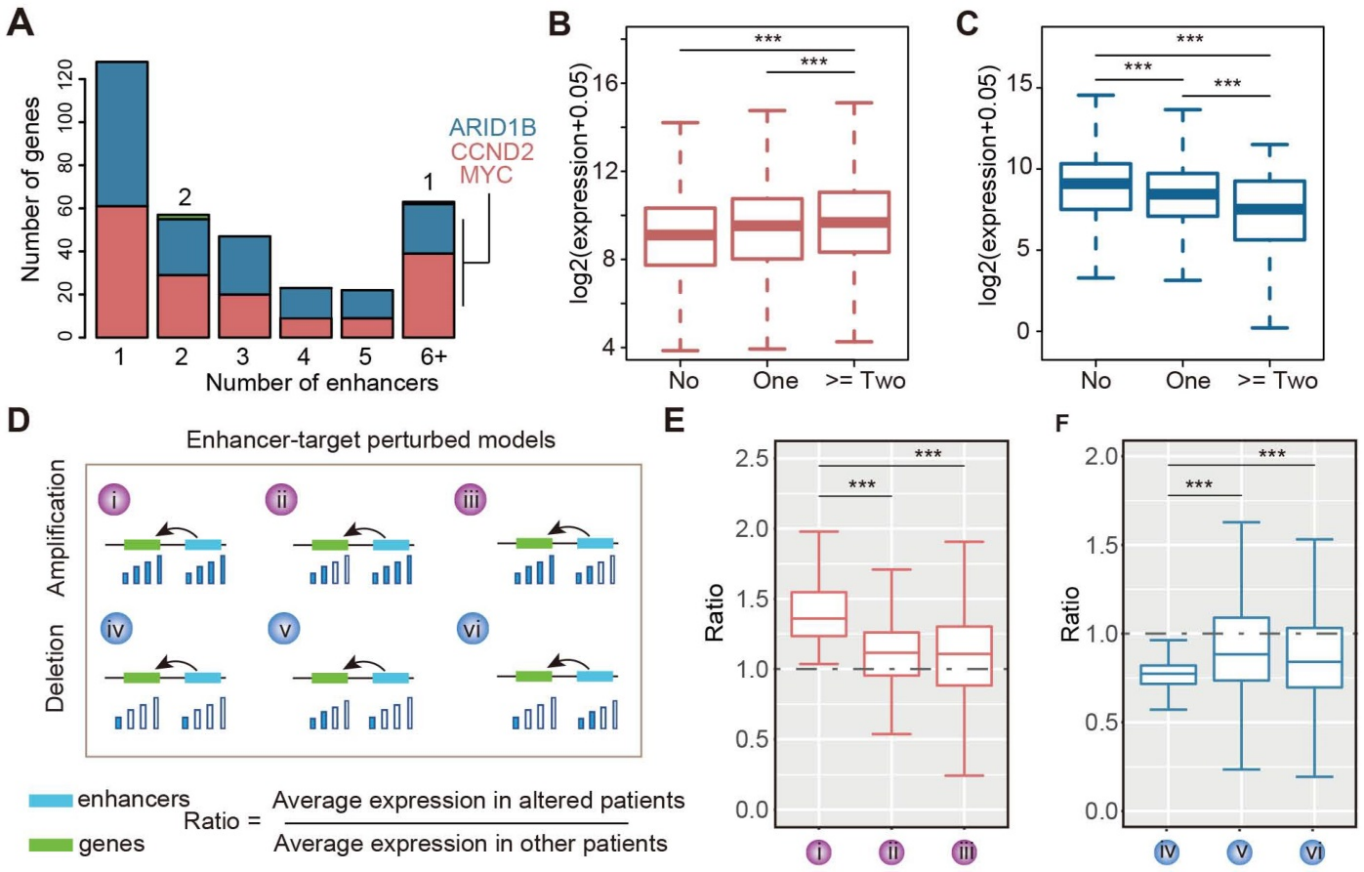
** CNAs and enhancers cooperatively regulate the expression of genes.** (A) Number of genes regulated by different number of enhancers in glioma. (B) The expression of genes regulated by different number of amplified enhancers. (C) The expression of genes regulated by different number of deleted enhancers. (D) Regulatory models proposed for CNAs and enhancers cooperative regulation. (E) The expression differences for genes in different amplified models. (F) The expression differences for genes in different deleted models.

We next calculated the number of enhancers regulating each target gene. Interestingly, we found that approximately 62.4% of target genes were associated with two or more enhancers (Figure 3A and Figure S6A). This result suggested that multiple enhancers could cooperatively regulate the expression of target genes. Particularly, MYC, CCND2, and ARID1B were regulated by more than six enhancers in gliomas. We obtained similar results for different grades of glioma (Figure S6B-D). Next, we investigated the expression of genes regulated by a different number of enhancers. We found that the expressions of genes regulated by amplified enhancers were higher than those not regulated by enhancers (Figure 3B). Moreover, genes regulated by more than two amplified enhancers exhibited significantly higher expression in patients with glioma (Figure 3B). By contrast, genes regulated by deleted enhancers exhibited significantly lower expression, particularly for genes regulated by more than two deleted enhancers (Figure 3C). These results suggested that CNVs of enhancers might cooperatively regulate the expression of genes.

In addition, considering that the expression of target genes can also be affected by CNVs, we proposed six regulatory models for a given enhancer-gene regulation (Figure 3D). The CNA of both enhancers and genes may cooperatively regulate the expression of target genes. To validate this hypothesis, we investigated the expression of genes regulated by enhancers and CNAs. For the amplified group, we found that genes exhibited significantly higher expression in enhancer- and gene-amplified patients than in only enhancer or only gene-amplified patients (Figure 3E). Moreover, both enhancer- and gene-deleted patients exhibited significantly lower expression of genes compared with enhancer- or gene-deleted patients (Figure 3F). These results suggest that CNAs of enhancers and genes cooperatively regulate the expression of target genes.

### Enhancer-TF-target regulatory circuit perturbations in glioma

**Figure 4:**
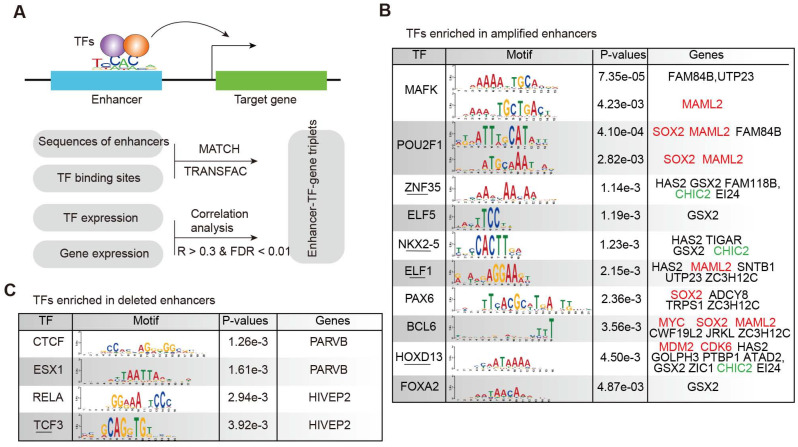
** TF**-**enhancer**-**gene regulatory circuits in glioma.** (A) The computational model for identifying enhancer-TF-gene triplets in glioma. (B) TFs enriched in amplified enhancers. (C) TFs enriched in deleted enhancers.

As active enhancers need to bind TFs to regulate downstream gene expression 45, 46, we identified the TF-target regulation associated with enhancers to understand the regulatory circuit during glioma malignant progression. Integrating the TF-enhancer pair, enhancer-target gene regulation and the expression of TFs and genes, we identified the enhancer-TF-target triplets in glioma (Figure 4A). Based on the sequences of amplified enhancers, we found that the binding sites of MAFK, POU2F1, HOXD13, and FOXA2 were significantly enriched (Figure 4B). MAFK is a nerve growth factor, responsive immediate early gene that regulates neurite outgrowth 47. PAX6, BCL6, HOXD13, and FOX2 have also been shown to be associated with glioma in previous studies 48-50. For the deleted enhancers, we identified four TFs significantly binding to the enhancer sequences (Figure 4C), including CTCF, ESX1, RELA and TCF3. CTCF has been found to directly participate in chromosome architecture and is involved in forming the loops between the binding sites to further affect the enhancer-promoter interaction 51. TCF3 (Transcription factor 3) is a member of the T-cell factor/lymphoid enhancer factor (TCF/LEF) family, and high levels of TCF3 in glioma potentially promote glioma development through the Akt and Erk pathways 52.

**Figure 5:**
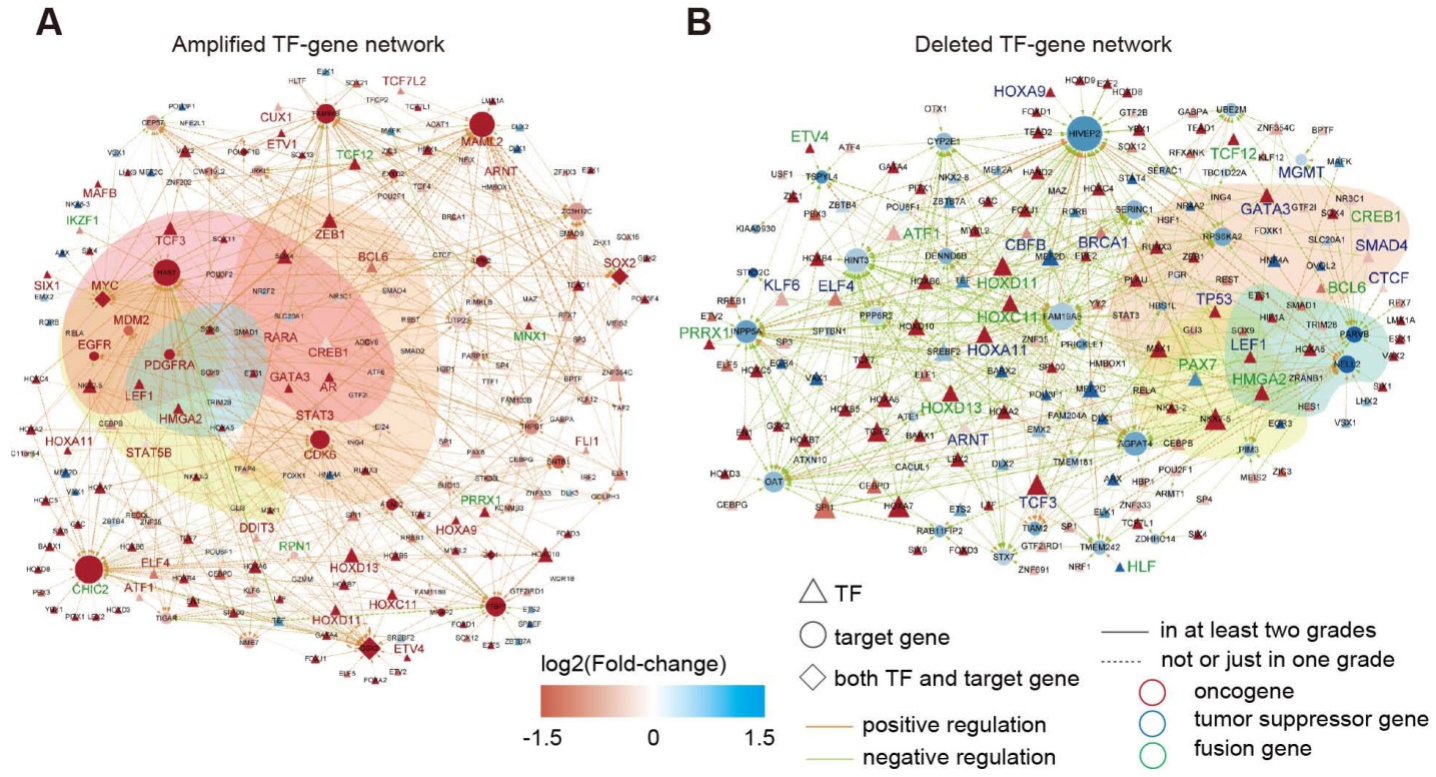
** TF-gene regulatory network in glioma.** (A) TF-gene regulatory network for amplified enhancers. (B) TF-gene regulatory network for deleted enhancers.

In total, we identified 545 TF-gene regulations associated with 141 enhancers with increased activity, and 469 TF-gene regulations were associated with 119 enhancers showing reduced activity (Tables S4-5). By integration of the TF-gene regulations, we constructed two regulatory networks in glioma (Figure 5A-B). Interestingly, we identified a number of oncogenes in the activity-increased network, whereas tumor suppressor genes were more frequently observed in the activity-reduced network. We next repeated this process for each grade of glioma. A number of interactions reappeared in at least two grades (Figure S7), suggesting that these interactions influenced the glioma in multi-grades. We next performed functional enrichment analysis and found that these genes were mainly associated with cancer hallmarks, such as “insensitivity to antigrowth signals”, “self-sufficiency in growth signals”, “tissue invasion and metastasis” and “evading apoptosis” (Figure S8).

Particularly, we found an amplified enhancer-SIX1-MYC regulation in glioma (Figure S9A). Based on the previous studies, Six1 is able to activate the expression of c-Myc 53. Both SIX1 and MYC were upregulated in patients with glioma with amplified enhancer. Moreover, the greater slope in the amplified group suggested that SIX1 might influence the expression of MYC more strongly in patients with amplified enhancer. Another example was the enhancer-CREB1-MGMT regulatory circuit (Figure S9A). CREB1 was upregulated while MGMT was downregulated in patients with deleted enhancers. Epigenetic silencing of MGMT by promoter methylation in glioma cells had been previously found 54. Here, our analysis provided novel insights into MGMT regulation in the context of the enhancer regulatory circuit. Moreover, we found that patients with glioma with distinct survival time can be effectively distinguished from each other based on the expression of TFs and genes (Figure S9B). Taken together, these results suggested that widespread enhancer-TF-gene regulatory circuit perturbations in glioma. Analysis of these regulatory circuits revealed critical genes in glioma.

### Core TF-target regulatory module associated with glioma prognosis

**Figure 6:**
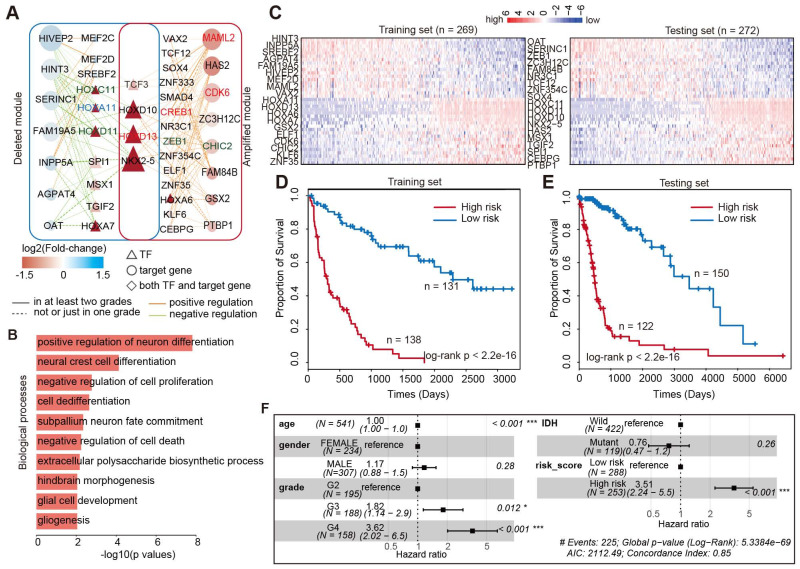
** Core TF**-**gene module associated with survival of glioma patients.** (A) Network view of the TF-gene regulation in core module. (B) Biological processes enriched by the core TF-gene module. (C) Heat maps showing the expression of TF and genes in glioma patients. Left is for training set and right is for testing set. (D) Kaplan-Meier estimates of the probability of survival based on the risk score model in training set. (E) Kaplan-Meier estimates of the probability of survival based on the risk score model in testing set. (F) Forest plot of multivariable hazard ratios.

The above examples suggested that integrating the TF-gene regulatory network would help in identifying the prognosis biomarkers in glioma. Thus, we next aimed to identify the core TF-target regulatory module associated with glioma prognosis. We first calculated the connectivity of TFs and genes in the glioma global network, and found that approximately 13.2% of TFs regulated more than 20 target genes in enhancer-amplified network and 9% in enhancer-deleted network (Figure S10A). Moreover, approximately 26% of target genes were regulated by more than seven TFs in enhancer-amplified network and 20% in enhancer-deleted network (Figure S10B). These results suggested that a TF may regulate multiple target genes and multiple TFs may work in concert to regulate the expression of a gene. By integration of TFs and genes with higher connectivity, we identified 58 interactions including eight genes and 18 TFs, thus involving 143 enhancer-TF-target triplets in enhancer-amplified network (Figure 6A). In addition, 58 interactions with seven genes and 14 TFs, involving 203 enhancer-TF-target triplets, were identified from the enhancer-deleted network (Figure 6A). Four TFs (TCF3, HOXD10, HOXD13, and NKX2-5) were identified in both networks. We found that a large number of TFs were encoded by HOX genes, which have been demonstrated to be associated with multiple cancer types 55-57. Functional enrichment analysis suggested that these genes were mainly involved in differentiation and proliferation-related functions (Figure 6B).

To evaluate the clinical significance of this network module, we next divided the patients equally into the training and testing sets. Based on the Cox proportional model, we identified that 38 genes were associated with patient survival in the training set (Figure 6C). In total, there were 18 protective factors and 20 risk factors (Figure S11). These genes exhibited dynamic expression across patients in the training and testing sets (Figure 6C). Next, we calculated the risk score for each patient by integrating the expression of these genes. The patients were divided into low-risk and high-risk groups based on the median of risk scores in the training set. We found that the patients in high-risk group exhibited poor survival in both the training and testing sets (Figure 6D and 6E, log-rank p < 2.2e-16). These results suggested that the risk-score may serve as a marker of glioma prognosis. Moreover, adjusted comparison was then performed by fitting a multivariate Cox proportional-hazards model, adjusted for potential confounders for patient survival, such as age, grade, gender, and IDH mutation. We found that the integrated risk score was an independent predictor of survival in patients with glioma (Figure 6F, Hazard ratio = 3.51, p < 0.001). Taken together, we identified a few dozen of new prognostic TF-target regulations in glioma, which increased our understanding of the transcription regulatory mechanism mediated by enhancer activity alteration in glioma.

### Validation of the core regulatory module in independent cohorts

**Figure 7:**
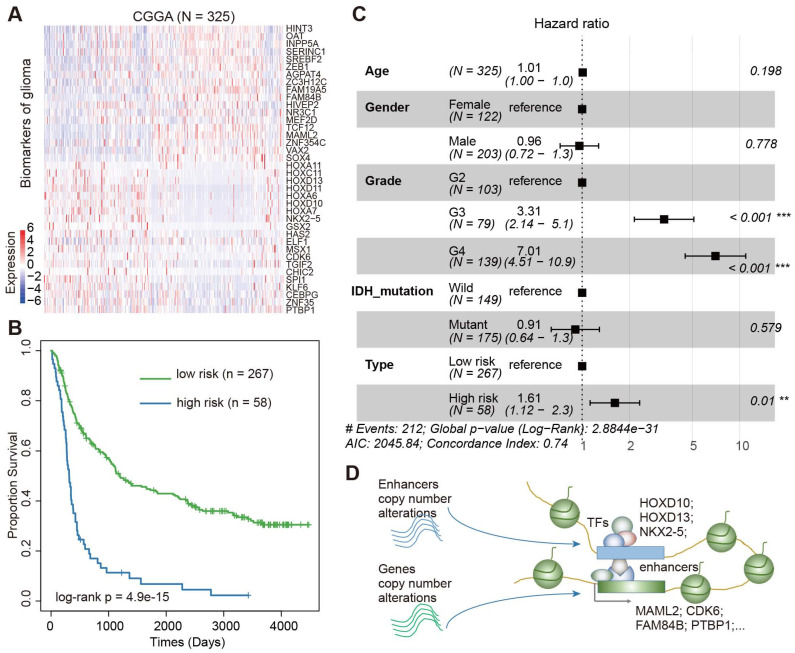
** Validation of Core TF**-**gene regulatory module in CGGA cohort.** (A) Heat map showing the expression of prognostic factors in CGGA cohort. (B) The Kaplan-Meier estimates of the probability of survival based on the risk score model in CGGA cohort. (C) Forest plot of multivariable hazard ratios in CGGA cohort. (D) Diagram showing the model for enhancer-centered TF-gene regulation driven by copy number alterations.

We next validated the prognosis effect of this core regulatory module in a Chinese cohort. In total, we downloaded the gene expression profiles of 325 Chinese patients with glioma from the Chinese Glioma Genome Atlas (CGGA) 58, 59. Visualization of the expression of these genes showed a great variation among the patients (Figure 7A). Based on the same model trained in the TCGA data, we divided the patients into low-risk and high-risk groups. We found significant differences in survival between patients in low-risk and high-risk groups (Figure 7B, log-rank p = 4.9E-15). Moreover, we performed a multivariate Cox proportional-hazards model, adjusted for age, grade, gender, and IDH mutation. We found that the integrated risk score was an independent predictor of survival (Figure 7C, Hazard ratio = 1.61, p < 0.01). Together, all these results suggested an enhancer-centered TF-gene regulatory model driven by CNAs in cancer. The expression of the core regulatory module is associated survival in patients with glioma.

### Enhancer target genes promote cell proliferation and migration in glioma

**Figure 8:**
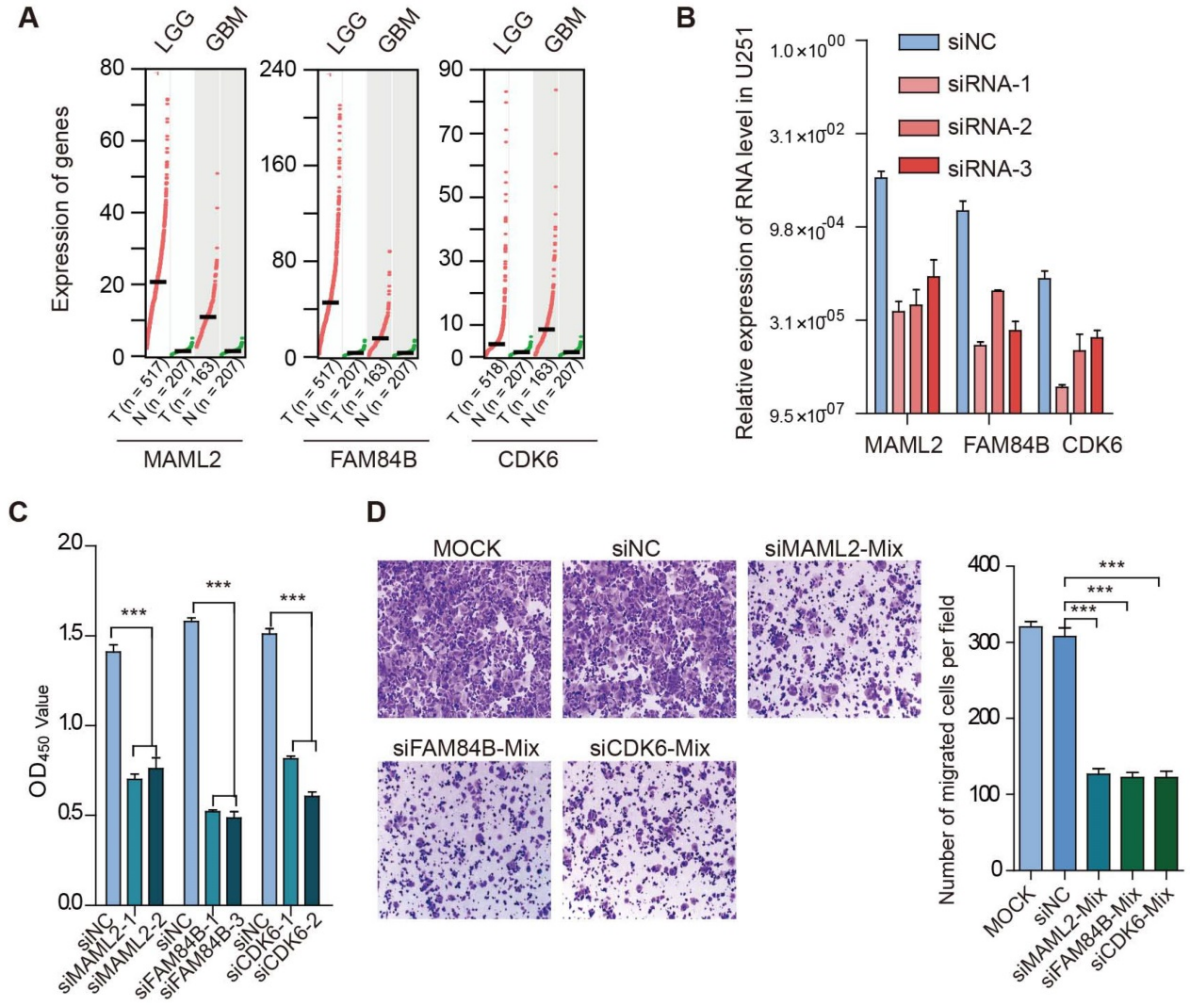
** Target genes promote cell proliferation and migration in glioma.** (A) Expression of genes in cancer and normal tissues. Normal tissues in TCGA and GTEx were used in this analysis. (B) Relative expression of genes in cell line. (C) Effects of target gene knockdown on glioma cell proliferation were measured by CCK-8 assays. (D) Representative images and bar graphs depicting the migration abilities of target genes-silenced glioma cells.

We found that TFs encoded by HOX genes recurrently appeared in glioma, and these TFs regulated critical genes (such as MAML2, CDK6, FAM84B, and PTBP1) (Figure 7D). Next, we explored the function of enhancer target genes in a glioma cell line. Compared with normal tissues, we found that MAML2, FAM84B, and CDK6 exhibited higher expression in glioma (Figure 8A). We next used siRNA technology to knock down these target genes in U251 cell line and measure the expression of genes. We found that siRNAs significantly reduced the expression of target genes (Figure 8B). Moreover, the knockdown target genes (MAML2, FAM84B, and CDK6) significantly inhibited the cell proliferation in glioma (Figure 8C, p < 0.001). Transwell assays were employed to explore whether cell migration was affected after silencing of enhancer target genes. We found that the knockdown of target genes significantly decreased cell migration compared with the control (Figure 8D, p < 0.001). Together, all of these results suggested that enhancer target genes significantly promote cell proliferation and migration in glioma.

## Discussion

The activity alterations of enhancers, a well-known class of distal regulatory element, control the expression of cancer-related genes and contribute to cancer pathogenesis in multiple cancer types 19. In this study, we identified robust enhancers by integration of the CAGE expression of enhancer RNA and genomic location. Through integration of TCGA copy number data, we identified numerous enhancers with activity alterations during glioma progression. Particularly, the activity alterations were much greater in high patients with high-grade glioma. Extensive studies have shown that CNVs not only directly affect the expression of genes but also regulate gene expression by acting on distal regulatory elements, such as enhancers 60. We, indeed, found an additive effect where the expressions of genes were affected by CNVs and by relevant enhancers. Moreover, the genes regulated by enhancers were enriched in cancer-related pathways.

The enhancers contain some recognition sequences that can specifically be bound by TFs that regulate gene expression in a spatial and temporal fashion 61. We also observed that activity alterations of enhancers perturb the regulation of genes by TFs. To identify prognosis-related TF-gene regulation in glioma, we identified a network module from the global transcriptional regulatory networks. The TFs encoded by HOX gene recurrently appeared, suggesting that this kind of TFs may play a crucial role in the occurrence and progression of glioma 62. These TFs regulate critical genes (such as MAML2, CDK6, FAM84B and PTBP1) that play fundamental roles in cancer. In addition, we found that several TF regulations, such as SOX4-MAML2, CREB1-CDK6 and NR3C1-FAM84B, were supported by ChIP-Seq in Cistrome DB 63. Finally, we identified several genes that potentially contributed to prognosis of glioma, which were further validated in an independent cohort. These potential prognosis genes and TF-gene regulation provide a new view on the future glioma therapy.

A detailed understanding of the molecular mechanisms of enhancer regulatory circuit perturbations still remains an important question. Given increasing evidence that the majority of disease-associated sequence variations are observed in enhancers, it is interesting to investigate whether these variations will alter the TFs binding 64. In addition to TFs, miRNAs, circRNAs 65, and RNA-binding proteins (RBPs) constitute another key layer in the maintenance of gene expression 66-68. Surprisingly, relationships between enhancers and miRNAs or RBPs have not been studied 69. The further exploration of miRNA or RBP regulation of enhancer transcription will therefore be the central importance for understanding of function of enhancers.

In summary, our network-based analysis revealed enhancer-centered regulatory circuit perturbations during glioma progression and identified core module associated with patient survival. All these results provide new molecular insights into enhancer functions and may advance novel therapeutic interventions for gliomas.

## Supplementary Material

Supplementary figures.

Supplementary table 1.

Supplementary table 2.

Supplementary table 3.

Supplementary table 4.

Supplementary table 5.
