# Supplementary material for: Systematic analysis of enhancer regulatory circuit perturbation driven by copy number variations in malignant glioma
Source: Theranostics. 2021 Jan 1;11(7):3060–73. doi: 10.7150/thno.54150 (PMC7847679; doi:10.7150/thno.54150)
Supplement: Supplementary file 1 — Supplementary figures. [file thnov11p3060s1.pdf]

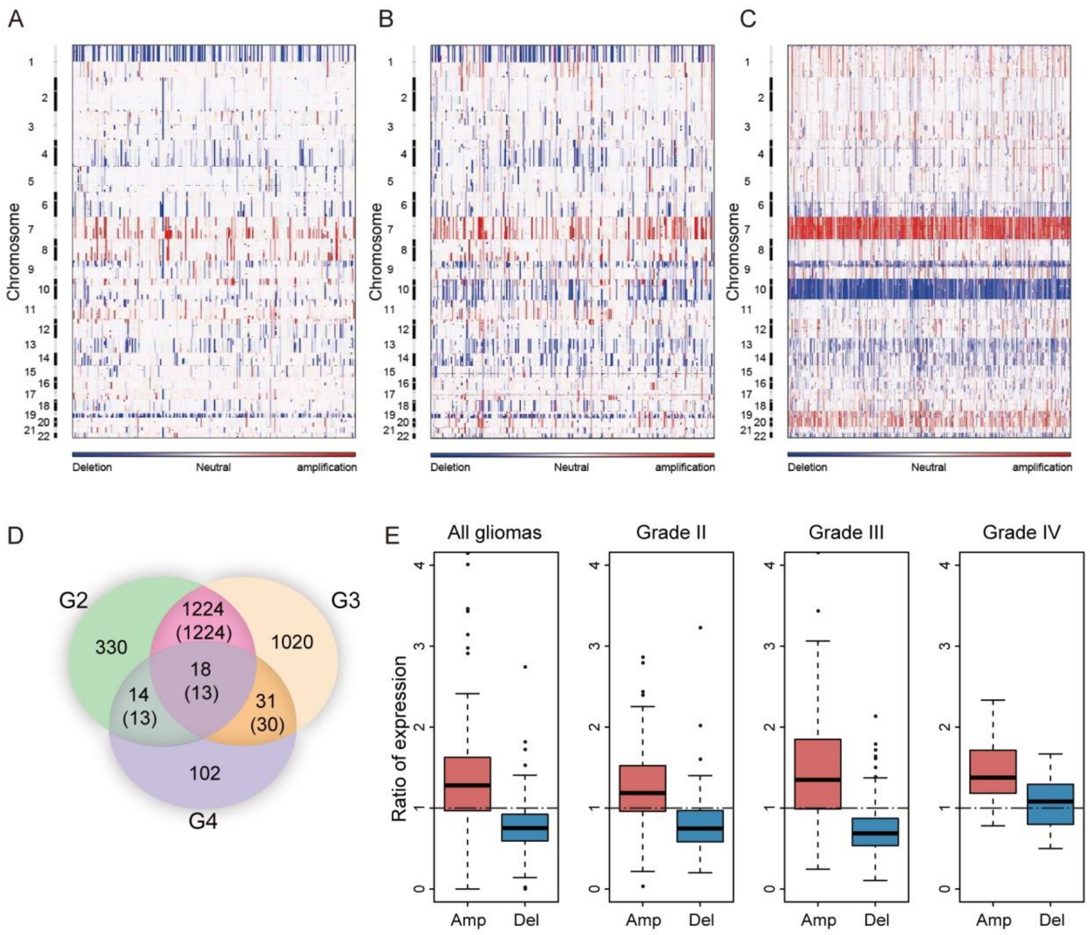

Fig. S1. CNVs of enhancers across different grades of glioma.
(A)-(C), Copy number values across different chromosomes in G2 (A), G3 (B) and G4 (C) grades glioma. (D), Venn plot showing the overlap of enhancers with CNVs. The numbers in brackets reveal enhancers with the recurrent amplification or consistently deletion. (E), The expression of differences for enhancers in enhancer amplified and deleted patients.

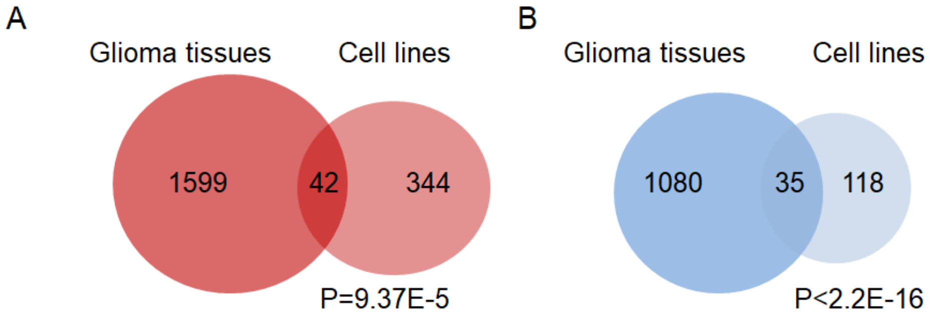

Fig. S2. Overlap of CNA-driven enhancers between glioma tissues and cell lines. (A) Overlap of amplified enhancers. (B) Overlap of deleted enhancers.

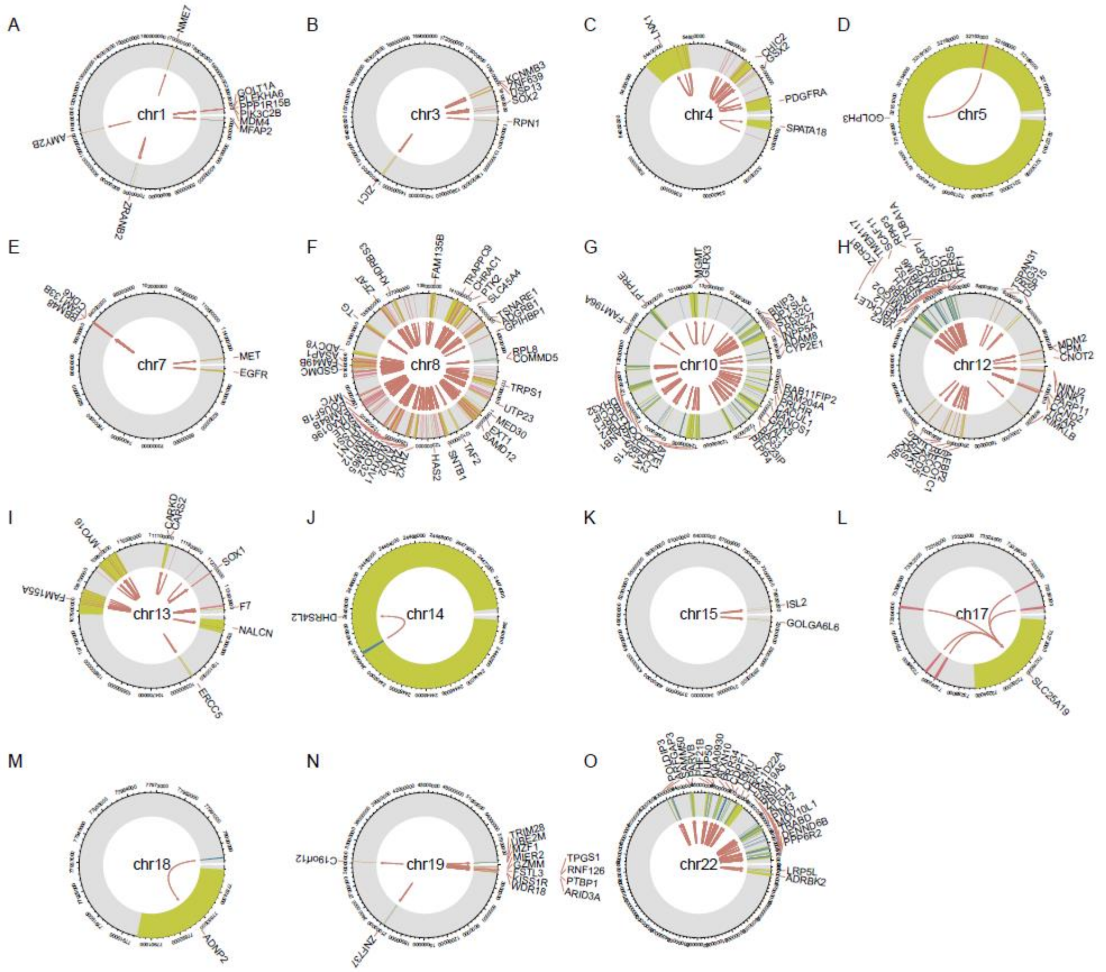

Fig. S3. Circos plots showing the enhancer-gene regulation in glioma. Red regions for enhancers with copy number amplification, blue regions for enhancers with copy number deletion, and green regions for genes regulated by CNV-driven enhancers. (A)-(O) for different chromosomes.

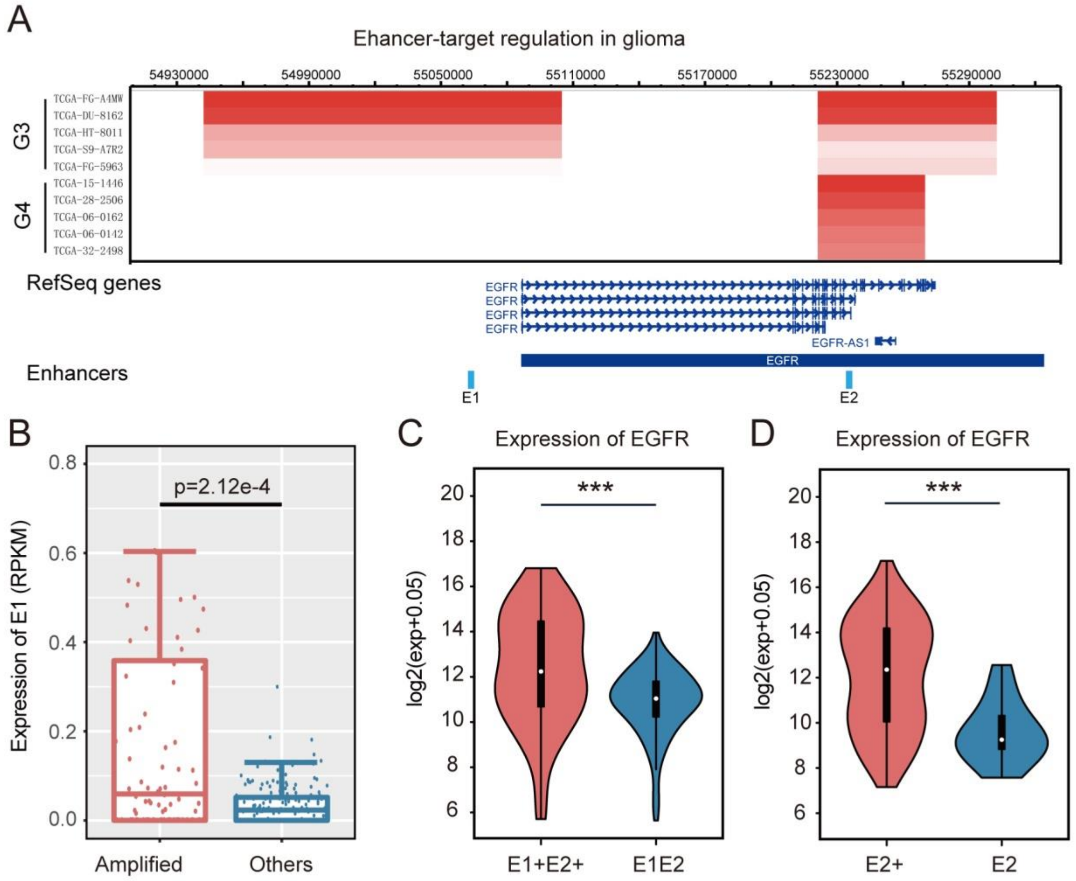

Fig. S4. Examples for enhancer-gene regulation in glioma.
(A), Track views of the enhancer-EGFR regulation in G3 and G4 glioma. (B), Boxplots showing the expression of E1 in amplified and other patients. (C), Boxplots showing the expression of EGFR in E1 and E2 amplified (red) or normal (blue) G3 patients. (D), Boxplots showing the expression of EGFR in E1 amplified (red) or normal (blue) G4 patients.

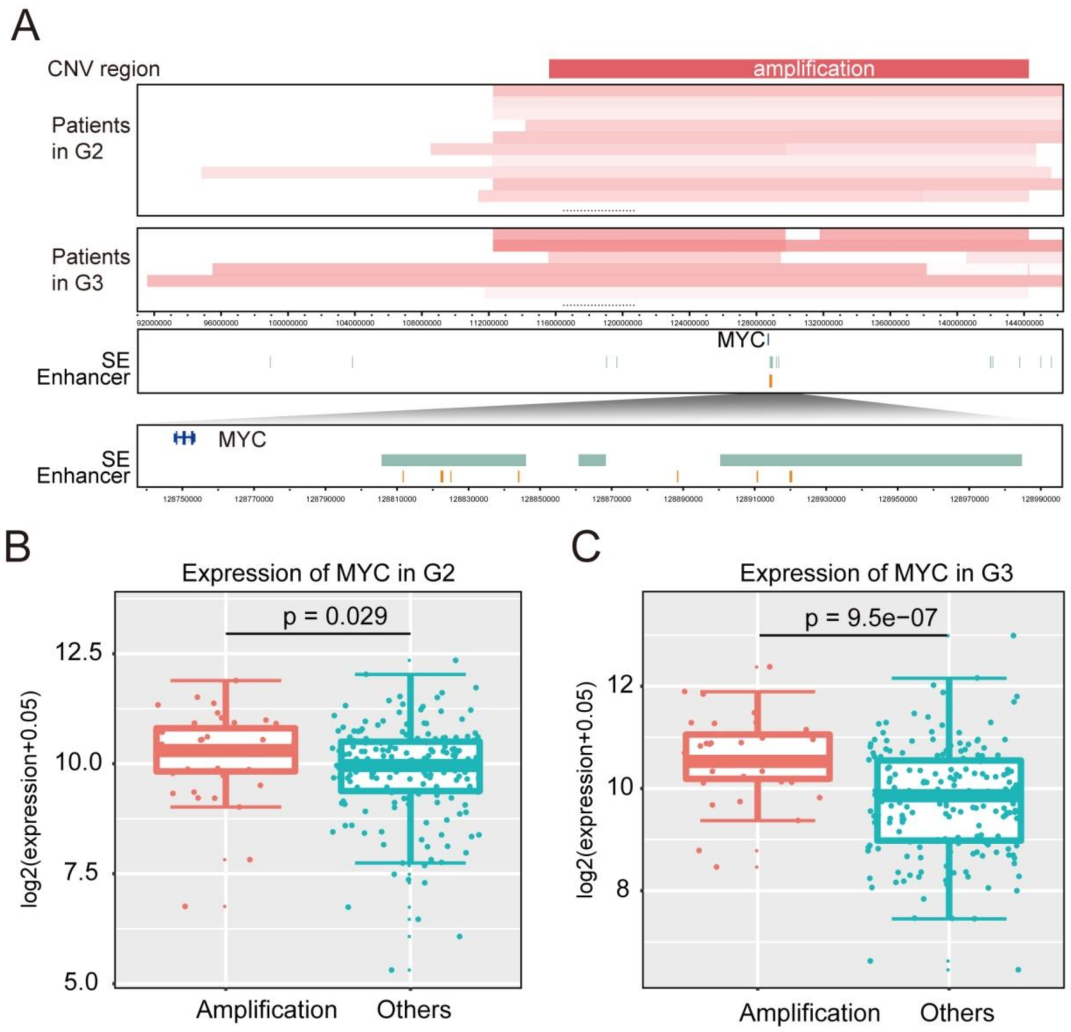

Fig. S5. Examples for enhancer-gene regulation in glioma. (A), Track views of the enhancer-MYC regulation in G2 and G3 glioma. (B), Boxplots showing the expression of MYC in enhancer amplified or normal G2 patients. (C), Boxplots showing the expression of MYC in enhancer amplified or normal G3 patients.

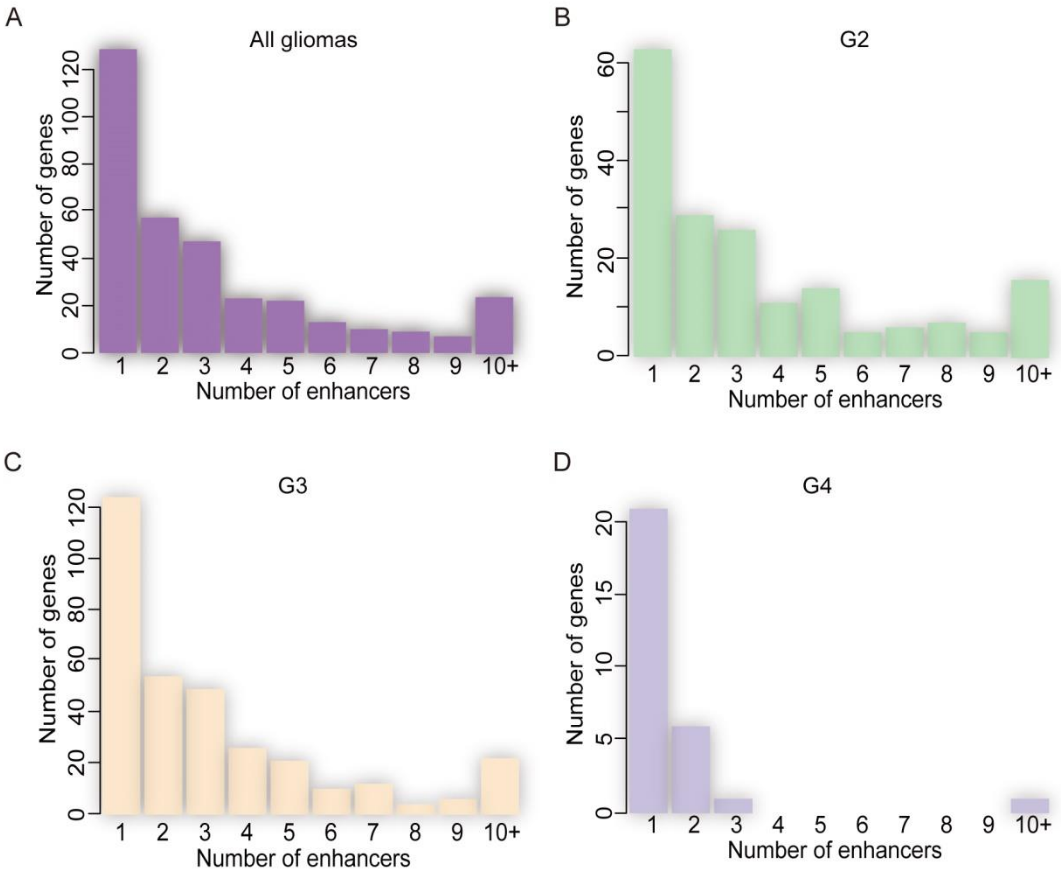

Fig. S6. Numbers of genes regulated by different numbers of enhancers. (A) for all glioma patients. (B) for G2 glioma patients. (C) for G3 glioma patients and D for G4 glioma patients.

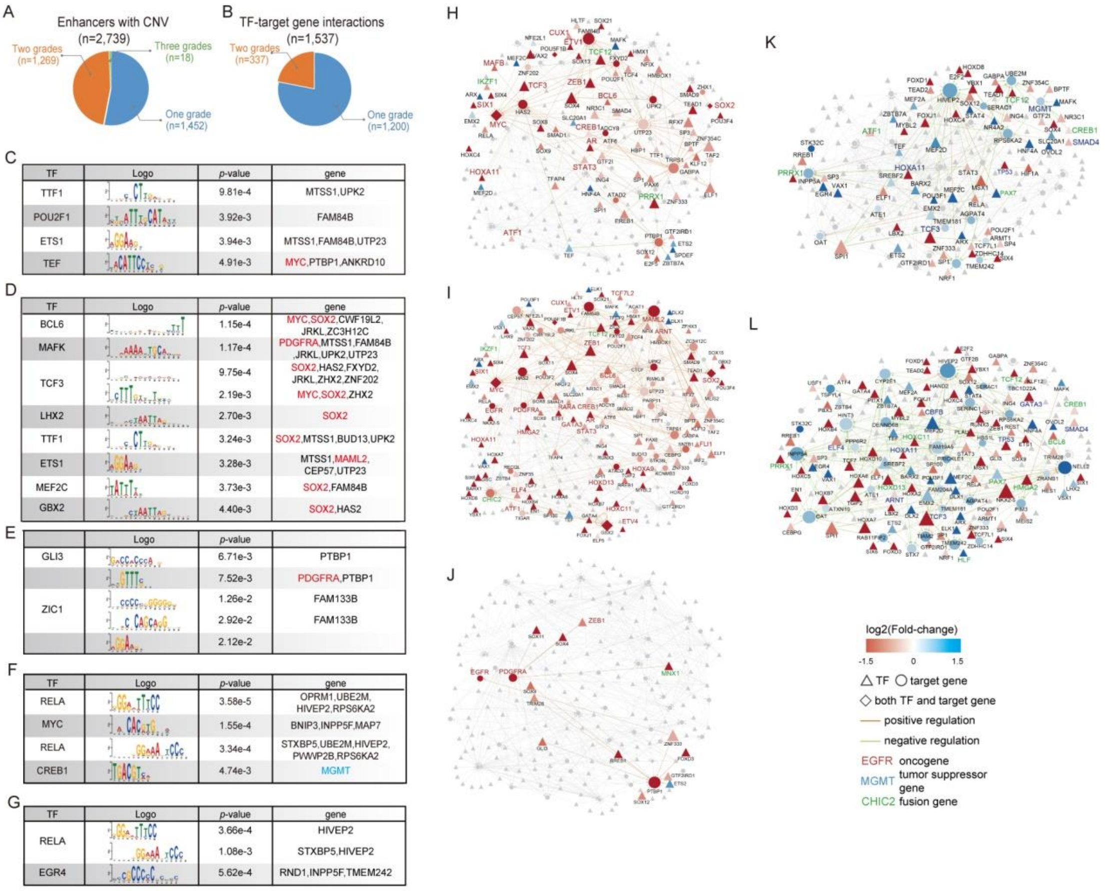

Fig. S7. Enhancer-TF-target triplets identified in each grade of glioma. (A), Pie chart showing the number of enhancers with CNV in different grades. (B), Pie chart showing the number of TF-target gene regulation in different grades. (C)-(E), The motif enrichment for amplified enhancers in enhancer-TF-target triplets in grade II (C), grade III (D) and grade IV (E). (F) and (G), The motif enrichment for deleted enhancers in enhancer-TF-target triplets in grade II (F) and grade III (G). (H)-(J), Global transcription regulatory networks mediated by amplified enhancers reproducing in grade II (H), grade III (I) and grade IV (J). (K) and (L), Global transcription regulatory networks mediated by deleted enhancers reproducing in grade II (K) and grade III (L).

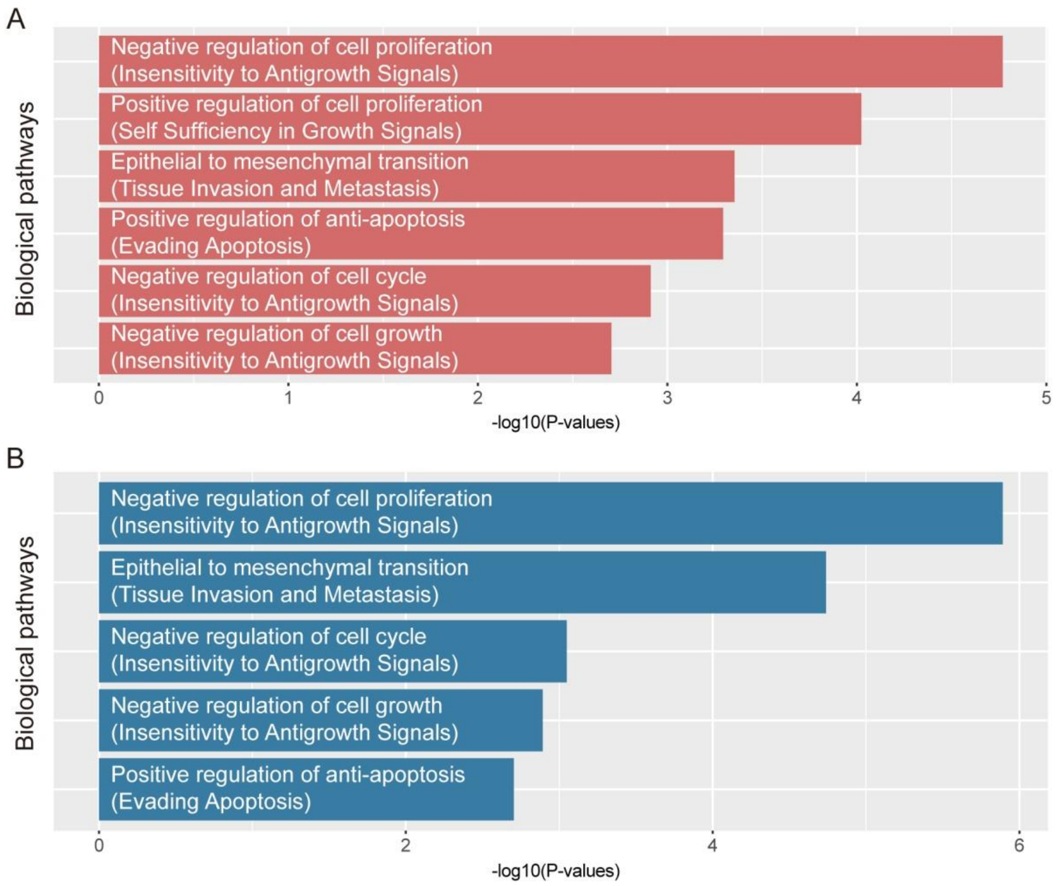

Fig. S8. Biological functions enriched by TF-gene regulations.
(A) for amplified enhancer-TF-gene regulation and (B) for deleted enhancer-TF-gene regulation.

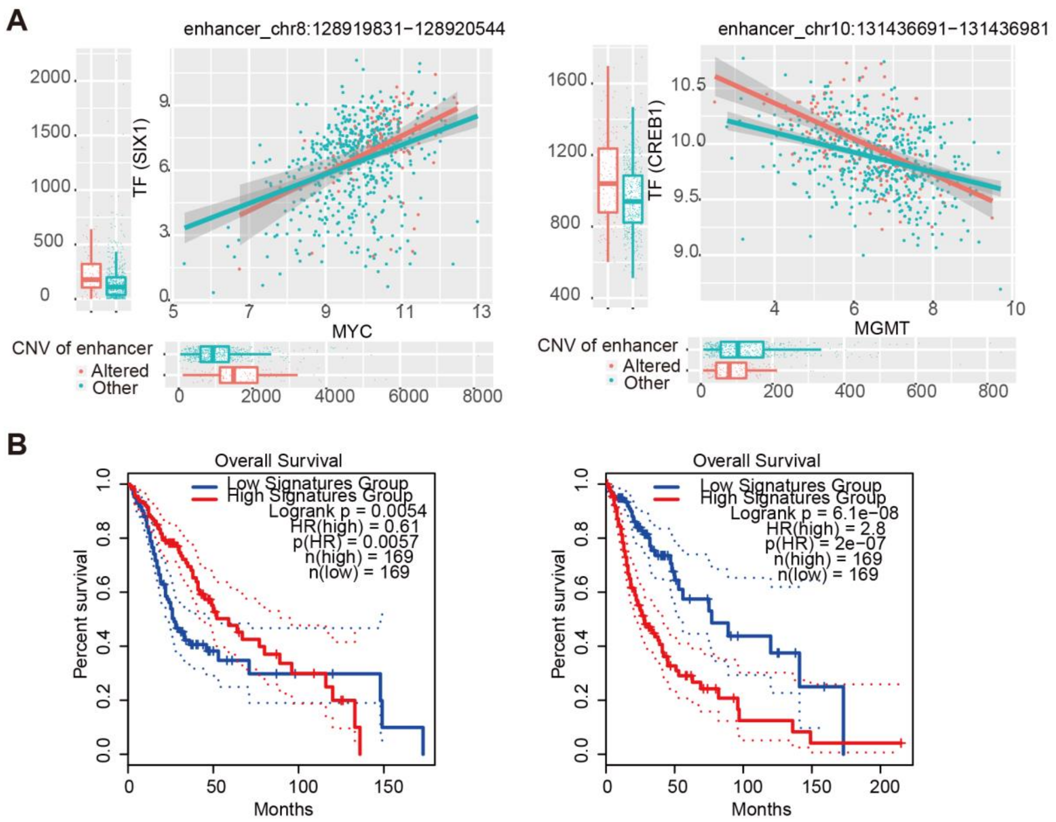

Fig. S9. TF-enhancer-target examples in glioma. (A) Boxplots showing distribution of TFs and genes expression in patients with or without enhancer altered. Scatter plots showing the correlation between the expressions of TFs and genes across glioma patients. (B) Kaplan–Meier estimates of the probability of survival based on the expression of TFs and genes.

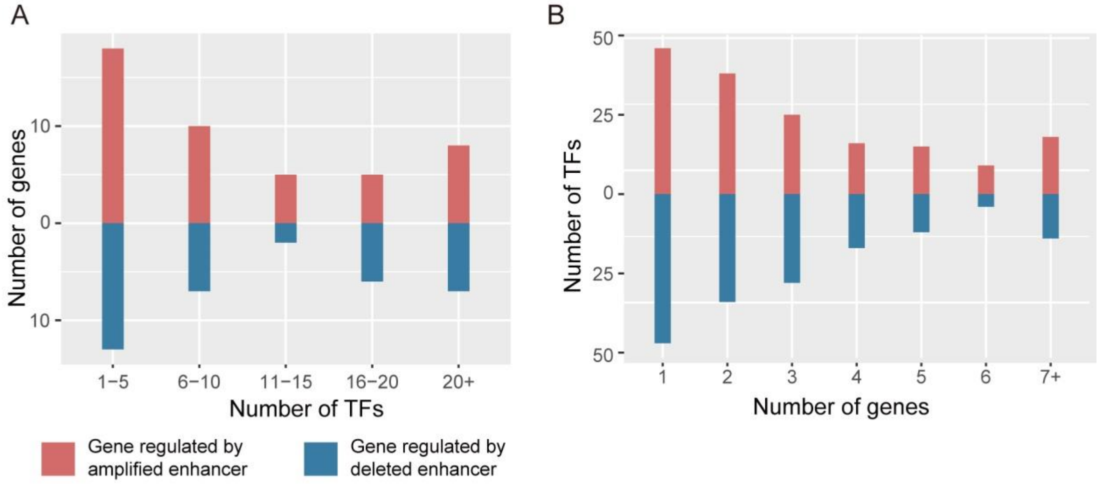

Fig. S10. Statistics for TF-gene regulation.

(A), Bar plots showing the numbers of genes regulated by different numbers of TFs. (B), Bar plots showing the numbers of TFs regulating different numbers of genes.

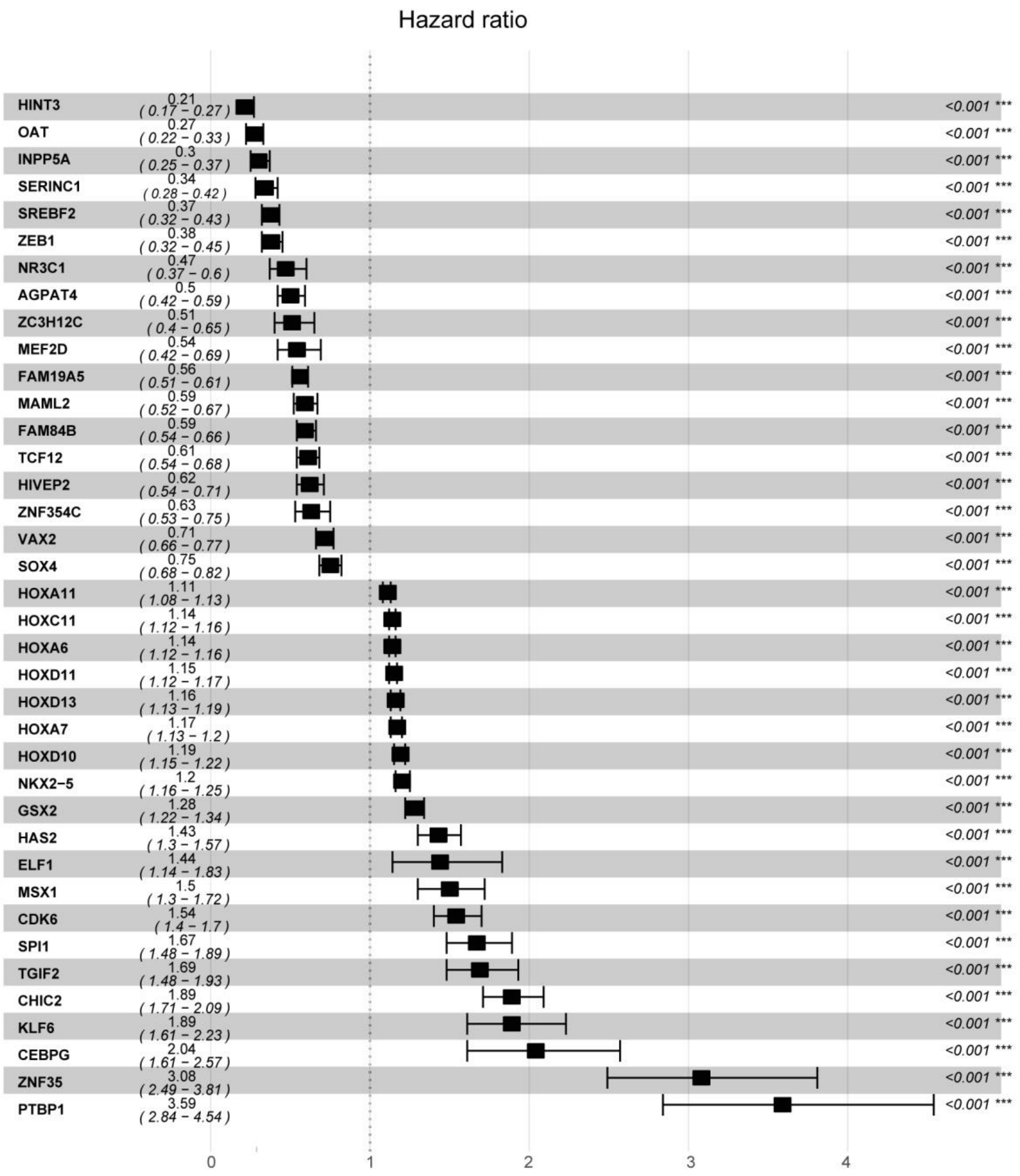

Fig. S11. Forest plot of univariate analysis based on expression of genes.
